# Potential role of the posterior cruciate ligament synovio-entheseal complex in joint effusion in early osteoarthritis: a magnetic resonance imaging and histological evaluation of cadaveric tissue and data from the Osteoarthritis Initiative

**DOI:** 10.1016/j.joca.2014.06.037

**Published:** 2014-09

**Authors:** D.A. Binks, D. Bergin, A.J. Freemont, R.J. Hodgson, T. Yonenaga, D. McGonagle, A. Radjenovic

**Affiliations:** † Leeds Institute of Rheumatic and Musculoskeletal Medicine, University of Leeds, Leeds, UK; ‡ Leeds NIHR Musculoskeletal Biomedical Research Unit, Leeds Teaching Hospitals NHS Trust, Leeds, UK; § Department of Radiology, Galway University Hospitals, Galway, Ireland; ‖ School of Medicine, University of Manchester, Manchester, UK; ¶ Institute of Cardiovascular & Medical Sciences, University of Glasgow, Glasgow, UK

**Keywords:** Knee OA, Synovitis, Enthesis, MRI

## Abstract

**Objective:**

This study explored posterior cruciate ligament (PCL) synovio-entheseal complex (SEC) microanatomy to determine whether it may participate in the early osteoarthritis (OA) disease process.

**Methods:**

SEC microanatomy and OA features were evaluated in 14 non-arthritic cadaveric knees (mean age = 69.9) using magnetic resonance imaging (MRI) and histology. MRI images of 49 subjects selected from the progression cohort of the Osteoarthritis Initiative (OAI) were evaluated by a musculoskeletal radiologist using an original semi-quantitative method for features associated with OA at the PCL tibial enthesis. Statistical analysis was performed using chi-square and Wilcoxon signed-rank tests to evaluate associations between SEC configuration and OA features.

**Results:**

The PCL formed a SEC-like structure encompassing bone- and ligament-lining intra-articular cartilages to which the posterior root of the medial meniscus contributed. Degenerative features at the PCL-SEC included: neovascularisation (44%), enthesis chondrocyte clustering (44%), collagen matrix fissuring at the enthesis (56%) and in the PCL itself (67%), tidemark duplication (44%), bone remodelling (44%) and microscopic inflammatory changes (33%). In the OAI cohort, SEC-related pathology included bone marrow lesions (BMLs) (69%) and osteophytosis (94%) at locations that corresponded to SEC-related cartilages. Posterior joint recess effusion (49%) was linked to MRI abnormalities at PCL-SEC cartilages (*χ*2 = 7.27, *P* = 0.007).

**Conclusions:**

The PCL has a prominent SEC configuration that is associated with microscopic OA changes in aged clinically non-diseased joints. MRI determined knee OA commonly exhibited pathological features at this site which was associated with adjacent joint effusion. Thus, the PCL-SEC could play a hitherto unappreciated role in the early OA disease process.

## Introduction

The pathogenesis of joint inflammation in osteoarthritis (OA) has consistently been conceptualised in relationship to articular cartilage damage with secondary synovitis^1^, ^2^. The importance of synovitis in OA is underscored by the fact that its presence is associated with both pain and more rapidly progressive joint destruction^3^ as assessed by arthroscopy^4^, magnetic resonance imaging (MRI)^5^, ^6^, ^7^, ^8^, ^9^ and C-reactive protein measurement^10^.

Synovitis and associated joint effusion in OA may have a localised distribution influenced by concomitant intra-articular pathology originating in adjacent structures such as the posterior horn of the medial meniscus^11^. To better explain the pathophysiological phenotypes of OA, we have used conventional and high resolution MRI and histological assessment of joints and have provided data relating to the importance of ligaments and tendons and their entheses as potential drivers in the pathogenesis of OA^12^. Combined high-resolution MRI and histological studies have previously demonstrated the involvement of ligaments and tendons in the early stages of hand OA^14^, ^13^. Other groups have shown evidence of anterior cruciate ligament (ACL) degradation and observed evidence of early histological changes in the ligament at various stages of macroscopic cartilage damage^15^.

It is now known that ligament attachments often form a complex anatomical functional unit involving the ligament itself as well as adjacent synovium and bony tuberosities^16^. It has also been noted that ligament and tendon insertions are not simply focal attachments that have fibrocartilage at the point of bony insertion, but the fibrocartilages extend into the immediately adjacent joint cavities to minimise stressing, forming structures termed synovio-entheseal complexes (SECs)^1^, ^17^. The SEC formed by the posterior cruciate ligament (PCL) at its tibial insertion includes the posterior horn of the medial meniscus which acts as a sesamoid fibrocartilage, and has been described briefly in the context of spondyloarthritis^16^.

The PCL is a strong structure that rarely ruptures and is not considered to be an important factor in the pathogenesis of knee OA in comparison to the ACL^18^. Recently, the anatomical distribution of synovitis and joint effusion associated with knee OA has been reported as being more common adjacent to the PCL^19^. A possible role for the PCL-SEC as an unappreciated contributor or driver of joint inflammation in this location has not been studied and remains unknown. The purpose of this study was to explore the PCL-SEC in non-arthritic cadaveric tissue and on MRI in OA subjects to determine whether it could contribute to the pathophysiology of OA. Here we use 3T MRI and correlative histology to show the involvement of the PCL-SEC in the early OA disease process.

## Methods

### Combined high-resolution MRI and histopathology of cadaveric tissue

**Fig. 1:**
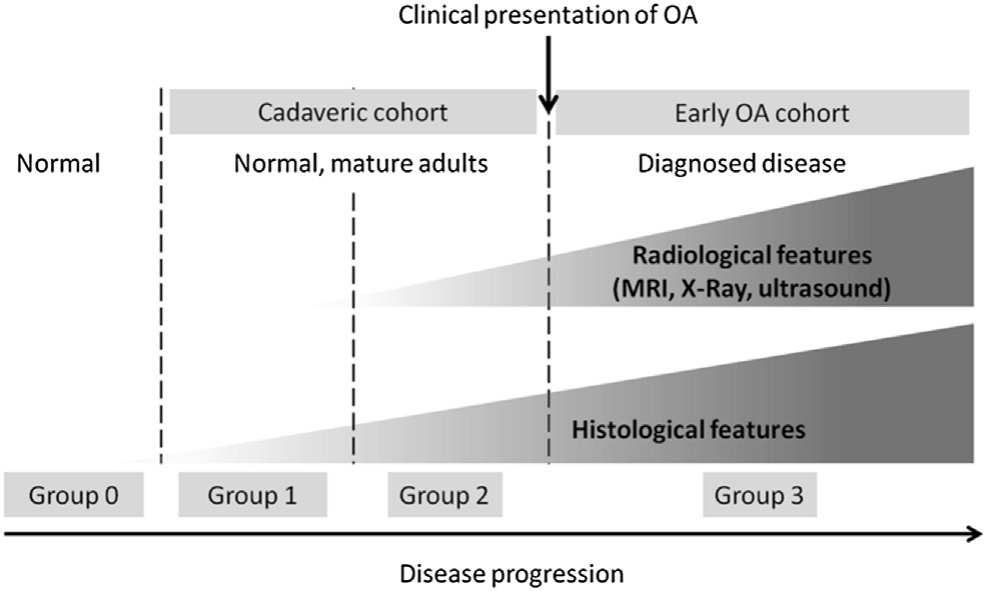
Categorisation of study cohorts with respect to clinical presentation of OA, radiological and histological features of disease. The cadaveric cohort in this study consists of samples taken from non-arthritic donors but in which some age related pre-clinical histological and radiological features were present (group 1 and group 2). Completely normal tissue is defined as that which lacks both histological and radiological features of disease (group 0) of which none were included in this study. The OAI cohort had clinically defined OA and is representative of group 3. This study focused on groups 1 and 2 (preclinical disease) and group 3 (clinically demonstrable disease). Accordingly there may be some overlap between these groups. Adapted from Binks *et al.* Ann Rheum Dis Published Online First: 4 October 2013 http://dx.doi.org/10.1136/annrheumdis-2013-203972.

Whole human knee joints were obtained from the Leeds GIFT Tissue Bank for the purpose of obtaining high-resolution MRI images of the tibial insertion of the PCL and comparative histopathology. The study was approved by the Local Research Ethics Committee and all donors had given their informed consent. Samples were collected from donors none of whom had an ante-mortem history of knee arthritis. A macroscopic assessment of tibial and femoral cartilage surfaces was made during dissection and no evidence of severe chondropathy was observed in any samples. For the purpose of the current study it is important to clarify what this might mean for disease pathogenesis. In this study our cadaveric specimens were taken from patients with no documented history of OA, but we did not obtain specimens from any young donors. Our specimens are therefore representative of normal, mature adults prior to clinical presentation, i.e., groups 1 and 2 depicted in Fig. 1.

**Fig. 2:**
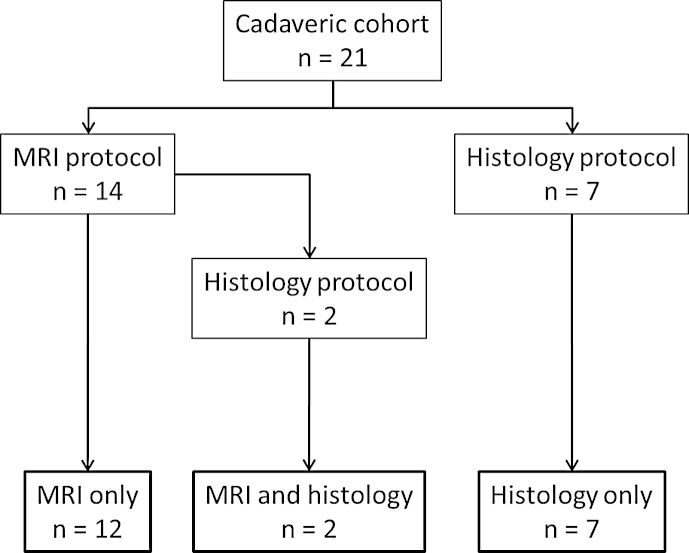
Flow diagram showing the protocols performed on the 21 knee joints comprising the cadaveric cohort.

21 knees were examined in total, from 18 donors (8 male, 10 female, mean age = 70.2 years). Whole joint MRI was performed on 14 cadaveric knees (6 male, 8 female, mean age = 69.9). Histopathologic analysis was performed on nine specimens (4 male, 5 female, mean age = 65.2), including two samples which had also undergone the whole joint MRI protocol. In summary, 12 samples underwent MRI analysis alone, seven samples underwent histological analysis alone and two samples underwent both histological and MRI analysis (Fig. 2). MRI images were acquired using a 3.0 T Siemens Verio system. The examination protocol implemented was based on that of the National Institute for Health (NIH) Osteoarthritis Initiative (OAI) MRI procedure for knee examination^20^, and has been reported previously^21^. Coronal intermediate-weighted (IW), 2-D turbo spin-echo (TSE) without fat-saturation, TR = 3700 ms, TE = 29 ms, FOV = 140 mm, matrix = 310 × 384; sagittal IW 2-D TSE with fat-saturation (FS), TR = 3200 ms, TE = 30 ms, FOV = 160 mm, matrix = 314 × 448; coronal T1-weighted, 3-D fast low-angle shot (FLASH) with water excitation, TR = 20 ms, TE = 7.57 ms, FOV = 160 mm, matrix = 512 × 512; sagittal 3-D dual-echo in steady state (DESS) with water excitation, TR = 16.3 ms, TE = 4.7 ms, FOV = 140 mm, matrix = 307 × 384 and; sagittal 2-D multi-echo spin-echo (MESE), TR = 2700 ms, TE = 10, 20,30,40, 50, 60, 70 ms, FOV = 120 mm, matrix = 269 × 384 sequences were performed. Coronal IW 2-D TSE (TR = 3850 ms, TE = 28 ms, FOV = 100 mm, matrix = 384 × 384, 8 signal averages) and sagittal IW 2-D TSE FS (TR = 3200 ms, TE = 36 ms, FOV = 160 mm, matrix = 512 × 512, 8 signal averages) were also performed with increased resolution.

Cadaveric MRI images were scored by a musculoskeletal radiologist with 11 years of experience (RJH) for the unequivocal presence of the following features: PCL full thickness tear; loss of continuous low signal line at the PCL insertion taken as corresponding to cortical bone disruption was evaluated using 3-D DESS and sagittal IW 2-D TSE FS sequences; high T2/fluid signal of fat in the region posterior to the PCL and/or soft tissue mass between PCL and fat corresponding to joint effusion was evaluated on sagittal IW 2-D TSE FS and sagittal 2-D MESE sequences; intraosseous cysts at the PCL insertion were noted and their largest diameter in the sagittal plane recorded using the 3-D DESS sequence. Two locations were assessed for the presence of osteophytes: (1) immediately posterior to the PCL insertion using 3-D DESS and sagittal IW 2-D TSE FS sequences and (2) immediately lateral to the PCL insertion using coronal IW 2-D TSE and 3-D FLASH sequences. The assessments were repeated by the same reader 2 weeks later with identical results. Integrity of the articular cartilage surfaces was assessed separately by another musculoskeletal radiologist with 8 years of experience (TY) using the whole-organ magnetic resonance imaging score (WORMS)^22^. Cartilage signal and morphology was evaluated in 14 defined sub-regions of the medial and lateral femorotibial joints and the patellofemoral joint using 3-D DESS and sagittal IW 2-D TSE FS sequences. Each region was scored on an 8 point scale (0–6). The maximum cartilage score for the whole knee was 84.

Following the MRI examination, the portion of the tibial plateau (TP) containing the PCL and ACL insertions was dissected retaining the first 2–3 cm of the anterior and PCL tissue proximal to the TP. Tissue samples were processed as described previously^21^.

The presence of a range of histopathologic features pertinent to the OA disease process were recorded and graded by one experienced pathologist (AJF) on a scale of 0–3, where 0 represented the absence of a feature and 3 represented a severe instance of the feature. The following features were scored in the PCL enthesis organ including changes at the insertion and adjacent SEC-related cartilages and synovium: intraligamentous calcification, matrix fissuring, chondrocyte clustering, chondrocyte hypertrophy, chondrocyte hypercellularity and hypocellularity, cartilage delamination, cell necrosis, cyst formation, neovascularisation, ligament/enthesis tear, vascular intrusion at cortical bone, tide mark duplication, bone remodelling, cartilage formation within the bone, inflammatory cell infiltration in the synovium, synovial cell hyperplasia, formation of synovial villi and synovial invasion of the enthesis.

### MRI of OAI patient cohort

MRI images of patients from the progression cohort of the NIH OAI study were assessed for the presence of PCL enthesis related pathology. Data used in the preparation of this article were obtained from the OAI database, which is available for public access at http://www.oai.ucsf.edu/. The specific dataset used was Image Release 0.B.2.

MRI images of the right knee were assessed in 49 patients (23 male, 26 female, mean age = 59.8, range 45–78). Subjects in the progression cohort of the OAI had at least one knee with radiographic OA defined as definite tibiofemoral osteophytes equivalent to a Kellgren and Lawrence grade of 2 or more and frequent knee pain or stiffness in the past 12 months.

PCL morphology and pathology was assessed in the 49 OAI MRI data sets by a musculoskeletal radiologist with 12 years of experience (DB) using an original scoring system and MRI images acquired at the enrolment visit of OAI. The presence of bone marrow lesions (BMLs) was noted using 3-D DESS and sagittal 2-D IW TSE FS sequences in three compartments, (1) at the site of the PCL attachment, (2) immediately anterior to the PCL attachment and (3) BMLs not at or immediately anterior to the PCL attachment. A scale of 0–3 was used to define severity of BMLs: 0 = no BMLs; 1 = BMLs 1 mm or less in depth from the cortex; 2 = BMLs greater than 1 mm but less than 10 mm from the tibial cortex; 3 = BMLs extending away from the tibial cortex greater than 10 mm. The integrity of the PCL fibres were assessed and graded from 0 (normal) to 3 (full thickness PCL tear). Cortical bone disruption at the PCL attachment was recorded (0 = not present, 1 = present). Tibial cartilage immediately anterior to the PCL enthesis was assessed and graded as normal or abnormal (including oedema and partial/full thickness cartilage loss). The presence of oedema, fluid and/or effusion in the posterior knee joint recess was recorded. The largest dimension in the sagittal plane of intraosseous cysts at the PCL tibial attachment were measured and the largest craniocaudal dimension of osteophytes posterior and lateral to the PCL tibial attachment were measured on sagittal and coronal plane images respectively. Chi-squared and Wilcoxon signed-rank statistics were calculated using SPSS v21 to test for differences among the dichotomously grouped variables. Results with *P* < 0.05 were considered to be statistically significant.

## Results

### PCL-SEC microanatomy

**Fig. 3:**
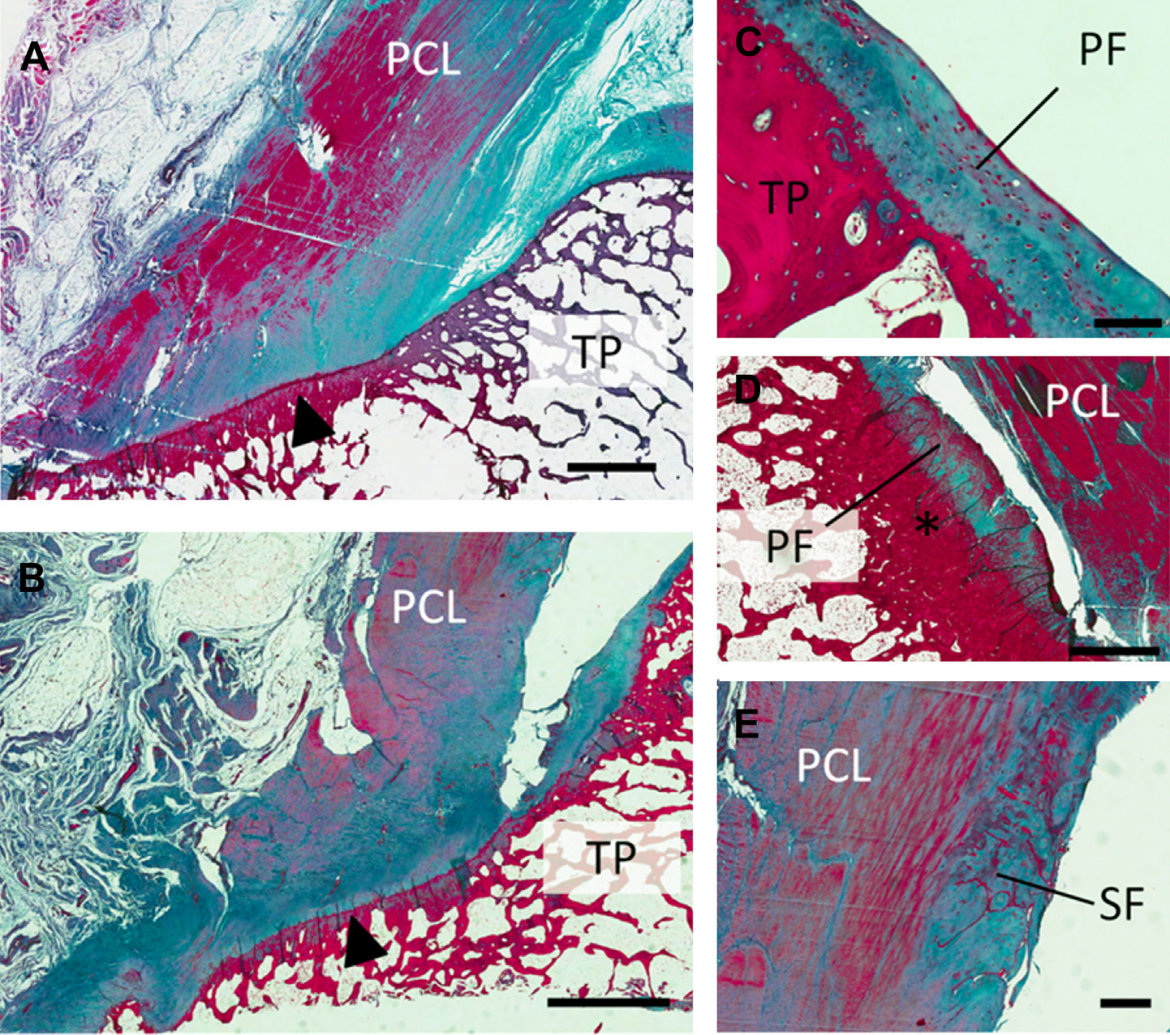
Sagittal histological sections stained with Masson's trichrome of the PCL attachment to the TP. (A) and (B), low magnification views of the posterior aspect of the TP and the PCL enthesis (arrowhead). Accessory (fibro) cartilages: (C) periosteal cartilage (PF) lining the surface of the posterior TP. (D) periosteal accessory cartilage continuous with the fibrocartilaginous attachment of the posterior horn of the medial meniscus adjacent to area of thickened bone (asterisk). (E) sesamoid accessory cartilage (SF) seen in the distal portion of the PCL. Scale bars, A, B and D = 2 mm, C = 100 μm, E = 500 μm.

Histological analysis of nine cadaveric specimens showed the anterior aspect of the PCL enthesis exhibited the characteristic features of a fibrocartilaginous enthesis with distinctive zones of calcified and uncalcified enthesis fibrocartilage. The posterior aspect of the enthesis was predominantly fibrous. Together with the immediately adjacent posterior aspect of the TP, the PCL enthesis formed an enthesis organ with the tuberosity of the TP acting as a bony pulley. This area of bone was frequently thickened relative to the surrounding bone and variably lined with cartilage of either hyaline or fibrocartilaginous type [Fig. 3(C–D)]. These accessory periosteal cartilages merged imperceptibly with the fibrocartilaginous attachment of the posterior horn of the medial meniscus. We also observed accessory cartilages – known as sesamoid cartilages – on the underside of the PCL distal to its tibial insertion [Fig. 3(E)]. These enthesis related “adaptive” cartilages correlated with areas of underlying bone thickening, likely equating with increased regional compressive loading. These intra-articular cartilages were intimately linked to posterior joint synovial reflections.

### Microanatomical damage in the PCL-SEC

**Fig. 4:**
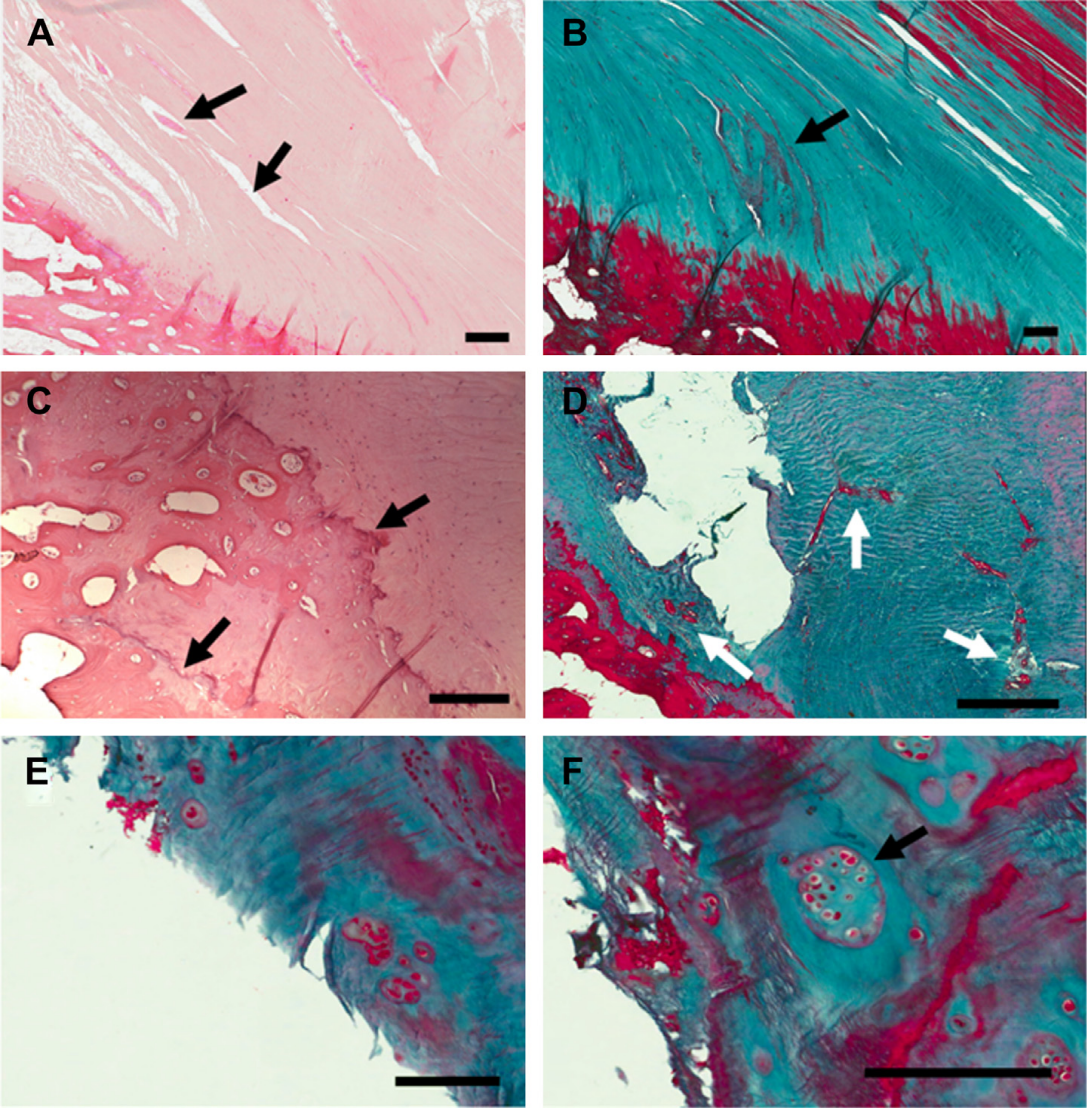
Microanatomical damage observed in histopathologic analyses of the PCL-SEC. (A) Fissuring of the collagen matrix (arrows) in the PCL is a feature commonly observed in the samples we studied. (B) Accumulation of myxoid material (arrow) in the fissured collagen matrix. (C) Tide mark reduplication (arrows) at the site of insertion was a common observation. In this case there is new bone formation in between the tide marks and evidence of cell necrosis. (D) Extensive neovascularisation (arrows) indicative of a reparative process observed at the PCL enthesis. (E) Fibrillation of the cartilage surface and (F) chondrocyte clustering (arrow) observed in the accessory sesamoid fibrocartilage of the PCL-SEC, both features typically associated with OA. Scale bars, A and B = 500 μm, C–F = 200 μm.

The PCL-SEC had numerous histopathologic features typically associated with OA (Fig. 4). In the enthesis and immediately adjacent portion of the ligament, including the accessory cartilages detailed above, we observed examples of intraligamentous calcification, matrix fissuring, chondrocyte cell clustering, cell hypertrophy and focal hypercellularity, cell necrosis, cyst formation, cartilage delamination and neovascularisation, with at least one change noted in each sample (Table I). The most frequent features observed were clustering of chondrocytes, observed in 44% of cases at the enthesis and 33% of cases in the adjacent portion of the PCL, matrix fissuring (56% enthesis, 67% adjacent PCL) and neovascularisation (44%, 22%). Neovascularisation was often seen in conjunction with other pathology indicative of tissue trauma. The neovascularisation was seen to weaken the ligament tissue as it cut through the collagen matrix and blood vessels were occasionally seen to empty into fissures in the matrix [Fig. 4(D)]. In some instances, we saw fissures in the collagen matrix with accumulated myxoid material resulting in expansion of the fissures [Fig. 4(B)]. In other cases matrix fissuring was associated with degenerative collagen fibres [Fig. 4(A)].

**Table I:** Frequency and grading of histopathologic features observed in the PCL tibial enthesis organ in nine non-arthritic cadaveric specimens. Features were graded on a scale of 0–3 where 0 represents an absence of the feature and 3 represents a severe instance of the feature

Histopathologic feature	Overall frequency	Grade 0	Grade 1	Grade 2	Grade 3
**Enthesis changes**					
Intraligamentous calcification	22%	78%	11%	11%	0%
Matrix fissuring	56%	44%	33%	11%	11%
Cartilage cell clustering	44%	56%	11%	33%	0%
Cell hypertrophy	33%	67%	11%	22%	0%
Hypercellularity	11%	89%	11%	0%	0%
Hypocellularity	11%	89%	0%	11%	0%
Fibrillation of cartilage	11%	89%	11%	0%	0%
Delamination of cartilage	33%	67%	22%	0%	11%
Necrosis	22%	78%	11%	11%	0%
Cyst formation	22%	78%	22%	0%	0%
Neovascularisation	44%	56%	33%	0%	11%
**Ligament changes (including accessory cartilages)**					
Intraligamentous calcification	22%	78%	22%	0%	0%
Matrix fissuring	67%	33%	11%	22%	33%
Cartilage cell clustering	33%	67%	11%	11%	11%
Cell hypertrophy	22%	78%	22%	0%	0%
Hypercellularity	22%	78%	11%	0%	11%
Hypocellularity	11%	89%	0%	0%	11%
Fibrillation of cartilage	11%	89%	11%	0%	0%
Delamination of cartilage	0%	100%	0%	0%	0%
Necrosis	22%	78%	11%	0%	33%
Cyst formation	33%	67%	22%	11%	0%
Neovascularisation	22%	78%	11%	0%	11%
**Synovium changes**					
Inflammatory cell infiltration	11%	89%	11%	0%	0%
Cell hyperplasia	11%	89%	11%	0%	0%
Synovial villi	22%	78%	22%	0%	0%
Synovial invasion of the enthesis	11%	78%	11%	0%	0%
**Other**					
PCL vascular intrusion at cortical bone	33%	67%	11%	11%	11%
Tide mark duplication	44%	56%	22%	11%	11%
Bone remodelling	44%	56%	22%	11%	11%
Cartilage formation within bone	33%	67%	22%	0%	11%
Enthesis tear	11%	89%	0%	11%	0%
Ligament tear	11%	89%	11%	0%	0%

Other prevalent arthritic features observed were vascular intrusion of the cortical bone at the PCL enthesis (33%), duplication of the “tide mark” at the junction of calcified and non-calcified cartilage [Fig. 4(C)] (44%) and cartilage formation within the bone (33%). The intra-articular accessory cartilages lining the posterior TP and the PCL were also subject to degenerative changes associated with OA. Fibrillation of the cartilage surfaces [Fig. 4(E)], chondrocyte proliferation [Fig. 4(F)] and neovascularisation were noted in at least one specimen examined.

Histopathologic changes in the synovium associated with the PCL enthesis were typically less severe and observed with lower frequency. Synovial changes of any sort were seen in 33% of the specimens examined. Mild (grade 1) inflammatory cell infiltration (predominantly lymphocytes), synoviocyte hyperplasia, synovial villi and synovial invasion were evident.

### MRI of cadaveric tissue

**Fig. 5:**
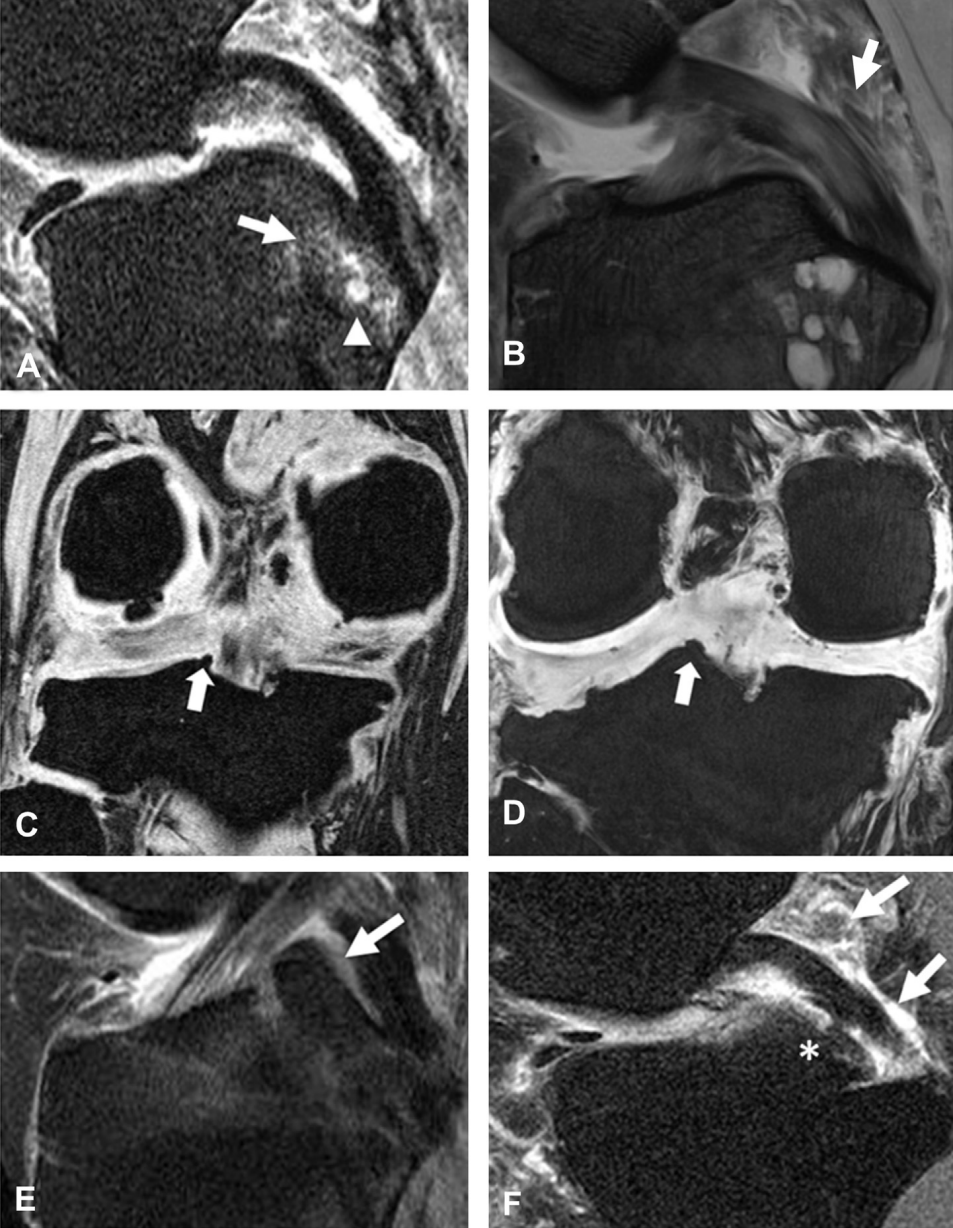
MRI observable pathology at the PCL-SEC in OAI participants and non-arthritic cadaveric tissue. (A) Sagittal 2D IW TSE FS image showing BMLs (arrow) and intraosseous cyst (arrowhead) observed in the regions adjacent and immediately anterior to the PCL insertion in a patient from the progression cohort of the OAI. BMLs were more frequently observed in the region immediately anterior to the PCL insertion. (B) Sagittal 2D IW TSE FS image showing intraosseous cysts observed in the same locations in cadaveric tissue and high signal in the fat posterior to the PCL compatible with joint effusion (arrow). Coronal T1W 3D FLASH images showing osteophyte formation (arrows) observed lateral to the PCL tibial insertion in OAI patient (C) and cadaveric tissue (D). (E) Sagittal 2D IW TSE FS image showing normal SEC cartilage (arrow) seen immediately anterior to the PCL tibial insertion in a patient form the OAI cohort. (F) Sagittal 2D IW TSE FS image showing high signal compatible with posterior recess joint effusion (arrows) which was found to be associated with abnormality in the SEC cartilage (asterisk).

The frequency of MRI lesions in clinically non-arthritic cadaveric knees, i.e., those with no ante-mortem history of OA, is summarised in Table II. Intraosseous cysts at the PCL were seen in 36% of cases, ranging from small individual cysts <5 mm in diameter to large cystic complexes >15 mm in diameter [Fig. 5(B)]. Osteophytosis in the region immediately lateral to the PCL tibial insertion – corresponding to the observed location of SEC cartilages – was another frequent observation [Fig. 5(D)] (57%) but was less common in the region posterior to the insertion (7%). High signal on T2 weighted images (oedema or fluid) in the posterior knee recess (posterior to the PCL) was seen in 64% of cases. The mean WORMS cartilage score of the 14 cadaveric knees was 16.0 (min = 0; median = 17; max = 54; standard deviation = 15.8). The maximum possible WORMS score was 84.

**Table II:** Frequency of ligamentous pathology features scored on MRI images of 49 knees from OAI and 14 non-arthritic cadaveric specimens

Ligamentous pathology feature	OAI cohort (*n* = 49)	Cadaveric cohort (*n* = 14)
Presence of BMLs at PCL attachment	33 (67%)	–
Presence of BMLs immediately anterior to PCL attachment	34 (69%)	–
Presence of BMLs not at or immediately anterior to PCL attachment	43 (88%)	–
Presence of BMLs at any site	46 (94%)	–
Full thickness PCL tear	0 (0%)	0 (0%)
Intraosseous cyst	25 (51%)	5 (37%)
Osteophytes posterior to PCL attachment	23 (47%)	1 (7%)
Osteophytes lateral to PCL attachment	46 (94%)	8 (57%)
Cortical disruption at PCL attachment	11 (22%)	1 (7%)
Posterior recess effusion	24 (49%)	–
Soft tissue mass in posterior knee recess	–	0 (0%)
High signal streaking in posterior knee recess	–	9 (64%)
Mean cartilage WORMS score (standard deviation)		16.0 (15.8)

### MRI of OAI progression cohort patients

In keeping with our observation of histological changes of bony thickening at the periosteal fibrocartilages in non-arthritic cadaveric tissue, in the cohort of OAI participants studied, PCL related BMLs were frequently observed in the region immediately anterior to the PCL insertion (69%) and also adjacent to the site of the insertion itself (67%) (Table II). Moreover, BMLs in the region anterior to the insertion were significantly more severe, with 31% of knees scored having BMLs graded 2 or 3 in this region compared to only 14% graded 2 or 3 in the region adjacent to the PCL insertion (Wilcoxon signed-rank test; *Z* = −2.14, *P* = 0.033). Intraosseous cysts at the site of the PCL attachment were present in 51% of the knees scored. Osteophytosis lateral to the PCL insertion, i.e., corresponding to the location of intra-articular SEC cartilages, was a common feature observed in 94% of knees [Fig. 5(C)]. Osteophytosis posterior to the PCL insertion was comparatively less common (*Z* = −4.80, *P* < 0.001) and was observed in only 47% of cases.

### SEC cartilage abnormality and association with posterior capsular effusion

High signal on fluid sensitive MRI sequences compatible with posterior capsular effusion occurred in 49% of knees. Furthermore, high signal in this region correlated with abnormality in the SEC cartilage immediately anterior to the PCL (*χ*^2^ = 7.27, df = 1, *P* = 0.007) [Fig. 5(E–F)], indicating an association between OA changes in SEC related cartilages and adjacent joint effusion.

## Discussion

This study investigated the microanatomical structure of the PCL-SEC and its tibial insertion point. In common with other entheses studied in detail previously^23^, the PCL enthesis and its adjacent synovium form a SEC structure^16^. The microanatomy of the PCL-SEC exhibits a number of features indicative of its involvement in the distribution and dissipation of mechanical forces. Areas of thickened cortical bone on the portion of the TP immediately anterior to the PCL insertion and the presence of periosteal fibrocartilage lining its surface and the associated sesamoid accessory cartilage on the immediately adjacent portion of the PCL evidence how these two structures undergo compressive loading as the knee joint articulates. A previous study has shown that fibrocartilages were subject to the full gamut of degenerative changes that are associated with articular cartilage pathology in OA^24^ and these degenerative changes were closely juxtaposed to immediately adjacent synovium which often exhibited micro-inflammatory changes, even in normal cases^23^.

In the present study, we also noted SEC-related fibrocartilages that were subject to the full extent of OA changes more usually associated with articular cartilage and frequently these indications of microtrauma were adjacent to regions of sclerotic bone or associated with fibrocartilaginous material, consistent with their origin being due to mechanical stimuli. Not only were these structures associated with microscopic synovitis in non-arthritic cadaveric joints and with evidence of OA changes on MRI in the same joints, but they were clearly linked to effusion in the posterior joint capsule in clinical OA.

There is controversy regarding the definition of age related normal changes and what is recognised as OA^25^, ^26^, and it appears that normality with respect to clinical OA sits along a pathological continuum. In this study, we selected cadaveric specimens based on the absence of a documented history of OA and macroscopically normal cartilage surfaces but these did not include any specimens from very young donors which would reasonably be expected to be totally free from histological and radiological changes. The continuum of such features observed in our cadaveric cohort can thus be interpreted as being representative of normal mature adults prior to the point of a confirmed diagnosis of OA.

The MRI analysis of patients from the OAI also showed a high prevalence of ligamentous pathologies in the region immediately adjacent to the PCL attachment. To the best of our knowledge this is the first time a detailed non-contrast enhanced MRI assessment of the PCL with histological correlation has been undertaken in the context of the OA disease process. Again, we believe these features are consistent with the localisation of compressive forces at the anterior portion of the PCL where the ligament presses against the adjacent tibial bone. This biomechanical influence also explains the distribution of both the radiological and histological features observed in our cadaveric cohort representative of mature adults prior to onset of diagnosed OA. In this cohort, the continuum of damage observed is associated with areas of high mechanical loading, as evidenced by the presence of accessory fibrocartilages. We postulate that in disease, this process is exaggerated and thus may be contributing to joint effusion in adjacent tissues.

The observed association between SEC cartilage abnormality and presence of posterior recess high signal on fluid sensitive MRI also offers some explanation in understanding the observed distribution of effusion posterior to the PCL as reported by Arden *et al.*^19^ It is conceivable that micro-debris from microanatomical damage in the PCL-SEC may be triggering a localised peri-ligamentous reaction with joint effusion in the posterior capsule region, in a manner identical to that which has been suggested for weight bearing articular cartilages^27^. Equally, the apparent functional integration of the SEC cartilages and posterior horn of the medial meniscus may also offer some explanation to the peri-meniscal synovitis associated with posterior meniscal damage as reported by Roemer *et al.*^11^ This work also links the location of SEC related cartilages to the observed distribution of osteophytosis within the knee joint whereby osteophyte formation occurs in the region adjacent to periosteal accessory cartilages associated with the PCL-SEC.

A limitation of the present study is that contrast-enhanced MRI sequences were not evaluated in the OAI patients for a proper assessment of synovitis. Fluid sensitive sequences were used to assess effusion which reflects synovial activation but effusion may sometimes be absent when synovitis is present^19^. A second limitation is that inter-/intra-observer reliability analysis was not performed for the MRI description of PCL ligamentous pathology. However, evidence of ligamentous pathology was observed in similar locations in both the cadaveric samples and in the OAI cohort and is still supportive of a possible link between PCL-SEC anatomy and OA pathology.

In conclusion we have demonstrated the PCL has features in common with previously identified SECs. Furthermore, we have observed microanatomical degenerative changes in the PCL-SEC of non-arthritic donors as well as the presence of MR observable macroscopic lesions. The association observed between abnormality in SEC cartilages and MRI pathology suggests the PCL-SEC plays an important role in the phenotypic expression of OA and these findings may have implications for the understanding of the OA disease process.

## Author contributions

All authors made substantial contributions to the study conception and design or analysis and interpretation of data and were involved in drafting the manuscript and approved the final version. Drs Binks and Bergin made equal contributions to this work. All authors take responsibility for the integrity of the work.

## Funding

This work was funded through WELMEC, a Centre of Excellence in Medical Engineering funded by the 10.13039/100004440Wellcome Trust and 10.13039/501100000266EPSRC, under grant number WT 088908/Z/09/Z.

## Competing interest statement

All authors declare that there are no conflicts of interest.
